# Stage-Specific Regulation of Ubiquitination Modifications and Prospects for Targeted Therapy in Triple-Negative Breast Cancer

**DOI:** 10.32604/or.2026.080113

**Published:** 2026-08-13

**Authors:** Yongpan Wang, Weiqiang Huang, Qizhuan Lin, Helei Cai, Fengjin Dai, Haiqing Gu, Shunyan Yu, Libo Jin, Renyi Peng

**Affiliations:** 1 Thyroid and Breast Surgery, The First People’s Hospital of Jiashan, Jiashan Hospital Affiliated to Jiaxing University, Jiaxing, China; 2 Institute of Life Sciences & Biomedicine Collaborative Innovation Center of Zhejiang Province, College of Life and Environmental Science, Wenzhou University, Wenzhou, China; 3 Huangqinyuan Branch, Tianshui Wulin Community Health Service Center, Gongshu District, Hangzhou, China

**Keywords:** Triple-negative breast cancer, ubiquitination, stage-specific regulation, targeted therapy, immune evasion

## Abstract

Triple-negative breast cancer (TNBC) is an aggressive subtype of breast cancer characterized by poor clinical outcomes. Owing to the absence of estrogen receptors, progesterone receptors, and Human Epidermal Growth Factor Receptor 2 (HER2) expression, TNBC shows limited responsiveness to conventional endocrine and targeted therapies. This subtype exhibits strong heterogeneity, a high propensity for metastasis, and a tendency to develop acquired drug resistance. Its survival and progression largely rely on non-classical signaling pathways, including Epidermal growth factor receptor (EGFR), Phosphoinositide 3-kinase/Protein Kinase B (PI3K/AKT), and Notch, which collectively impose substantial challenges to clinical management. In recent years, protein post-translational modifications—particularly ubiquitination and ubiquitin-like modifications—have emerged as key mechanisms governing TNBC malignant progression. This review systematically summarizes the stage-specific regulatory roles of ubiquitination during TNBC initiation, progression, and therapeutic resistance. These mechanisms include modulation of tumor stemness, epithelial–mesenchymal transition, DNA damage repair, ferroptosis, and immune evasion through the regulation of critical proteins such as Toll-like receptor 4/Nuclear factor kappa-B (TLR4/NF-κB), Twist family bHLH transcription factor 1 (TWIST1), Poly (ADP-Ribose) Polymerase 1 (PARP1), Programmed death-ligand 1 (PD-L1), and Glutathione Peroxidase 4 (GPX4). In addition, ubiquitin-like modifications (including small ubiquitin-like modifier (SUMOylation), Neural precursor cell expressed developmentally downregulated protein 8 (NEDDylation), and Interferon-stimulated gene (ISGylation)) function synergistically in maintaining genome stability, transcriptional regulation, and immunological processes. Furthermore, this review highlights the translational potential of targeting ubiquitination pathways in TNBC, covering the applications, advantages, and limitations of immune checkpoint inhibitors, Poly ADP-ribose polymerase (PARP) inhibitors, antibody–drug conjugates, and combination treatment strategies. Finally, it outlines future research directions, such as developing a TNBC-specific ubiquitinome landscape, creating highly selective E3 ligase or deubiquitinase inhibitors, and integrating multi-omics with artificial intelligence technologies. These advances are expected to provide a theoretical foundation and translational insights for precision interventions targeting protein homeostasis in TNBC.

## 1 Introduction

Triple-negative breast cancer (TNBC) is a breast cancer subtype characterized by the absence of estrogen receptor (ER), progesterone receptor (PR), and human epidermal growth factor receptor 2 (HER2) expression [1,2], accounting for 15%–20% of all breast cancer cases. Owing to the lack of these three classical treatment targets, TNBC exhibits distinct biological behaviors, clinical features, and treatment responses. It is generally more aggressive, associated with a higher risk of metastasis, and confers a poorer prognosis [3]. From a molecular pathological perspective, the loss of these receptors imparts unique tumor characteristics to TNBC [4]. Loss of ER disrupts estrogen-regulated growth mechanisms, leading to uncontrolled cell proliferation that is independent of endocrine regulation and renders tumor cells less responsive to therapy. Loss of PR not only reflects the non-functional state of the ER signaling pathway but is also associated with dysregulated cell cycle control and a higher proliferation index [5,6]. Loss of HER2 indicates that tumor cells are unresponsive to HER2-targeted therapies, while also enhancing sensitivity to exogenous growth factors, thereby promoting tumor invasiveness and metastasis [7]. The concurrent absence of these three receptors effectively establishes a “non-classical” proliferation network in TNBC, which bypasses traditional endocrine- or growth factor-driven signaling. Instead, TNBC relies on alternative “non-classical” pathways, including (Phosphoinositide 3-kinase/Protein Kinase B (PI3K/AKT): The key negative regulator of this pathway, Phosphatase and tensin homolog (PTEN), is frequently modulated by ubiquitination. E3 ligases (such as NEDD4 and WWP1) promote PTEN degradation through ubiquitination, thereby activating the PI3K/AKT signaling pathway [8,9]. Conversely, deubiquitinating enzymes (such as USP7 and USP11) stabilize PTEN and inhibit pathway activation. Additionally, AKT activity is also influenced by ubiquitination modifications, such as K63-linked ubiquitination, which promotes its membrane localization and activation), Epidermal Growth Factor Receptor (EGFR): EGFR is a commonly overexpressed growth factor receptor in TNBC. Ubiquitination, particularly K48-linked ubiquitination mediated by c-CBL, promotes EGFR internalization and degradation, thereby negatively regulating its signaling intensity [10,11,12]. In contrast, deubiquitinating enzymes, such as USP8 and USP22, stabilize EGFR by removing its ubiquitin chains, thereby enhancing downstream signaling. Similarly, the Notch receptor undergoes ubiquitination upon activation, a process mediated by E3 ligases including Itch and Nedd4, prior to cleavage by γ-secretase. This cleavage releases the Notch intracellular domain (NICD), which translocates to the nucleus to regulate transcription. Moreover, Notch receptor degradation is stringently controlled by the ubiquitin–proteasome system. In TNBC, aberrant activation of Notch signaling is closely linked to dysregulated ubiquitination, which supports tumor cell survival [13,14].

Compared with hormone receptor-positive or HER2-positive breast cancers, TNBC exhibits distinct molecular classifications, immune microenvironments, tumor metabolism, metastasis patterns, and mechanisms of drug resistance [15]. TNBC displays a high degree of heterogeneity and can be classified into multiple subtypes based on gene expression profiles, including basal-like, mesenchymal-like, immunomodulatory, and luminal androgen receptor (LAR) subtypes [16]. These subtypes show markedly different treatment responses and prognoses. In addition, TNBC is frequently characterized by an increased proportion of cancer stem cells, a strongly immunosuppressive microenvironment, active metabolic reprogramming, and epithelial–mesenchymal transition (EMT), all of which further exacerbate drug resistance and metastatic potential [17]. Although TNBC is relatively sensitive to chemotherapy in early stages, it is highly prone to relapse and rapidly develops multidrug resistance. Mechanisms underlying resistance include, but are not limited to, enhanced DNA damage repair (e.g., restoration of BRCA function), upregulation of multidrug resistance pumps, enrichment of cancer stem cells, and activation of anti-apoptotic pathways [18]. In recent years, targeted therapies directed at pathways such as EGFR, PI3K/AKT/Mechanistic Target of Rapamycin Kinase (mTOR), and Poly ADP-ribose polymerase (PARP) have shown potential. However, their overall efficacy remains limited due to heterogeneous target expression, variable treatment responses, and the high propensity for secondary drug resistance. Immunotherapy has shown promise in certain patients, particularly those targeting the programmed death-1 (PD-1)/programmed death-ligand 1 (PD-L1) axis inhibitors, which show relatively favorable efficacy in tumors with active immune microenvironments [19]. Nevertheless, the overall response rate remains low, and effective predictive biomarkers are still lacking.

In summary, TNBC cannot benefit from endocrine therapy or HER2-targeted therapy due to the absence of the three classical treatment targets: ER, PR, and HER2. The survival and progression of TNBC cells are largely dependent on non-classical signaling pathways, including EGFR, PI3K/AKT, Notch, and Wnt. The activation of these “independent” signaling mechanisms, combined with high molecular and immune microenvironment heterogeneity and abnormal metabolic reprogramming, collectively represents the core challenge in developing effective therapeutic strategies. Recent studies have indicated that protein post-translational modifications (PTMs) are key regulators of signaling pathways, metabolic homeostasis, and immune evasion, and have become a major focus in understanding TNBC drug resistance and invasiveness [20,21]. Among PTMs, ubiquitination directly modulates tumor cell sensitivity to radiotherapy and dynamically regulates lipid metabolism pathways by controlling the stability and degradation of critical signaling proteins [22]. For example, the ubiquitination status of proteins such as Cyclin-dependent kinase 8 (CDK8) and Fatty Acid Synthase (FASN) is closely associated with tumor proliferation and energy metabolism, playing a pivotal role in determining the response of tumor cells to exogenous damage. Abnormal regulation of the ubiquitination system also directly affects the stability of key oncogenic proteins [14,23]. Ubiquitination, one of the most prevalent post-translational modifications, plays a central role in regulating protein stability, cell cycle progression, DNA repair, and signaling pathway homeostasis. This process is mediated through the sequential action of the ubiquitin-activating enzyme (E1), ubiquitin-conjugating enzymes (E2), and ubiquitin ligases (E3), collectively referred to as the E1–E2–E3 cascade. Distinct ubiquitin chain linkages, such as K48- and K63-linked chains, determine the fate of substrate proteins, including their degradation, stabilization, or modulation of signaling activity. In TNBC, a highly aggressive and heterogeneous subtype, accumulating evidence suggests that dysregulation of ubiquitination constitutes a critical molecular mechanism driving tumor initiation and progression [24,25]. Ubiquitination regulation is closely linked to the characteristic molecular pathological features of TNBC, including dysregulated DNA damage repair pathways, maintenance of stemness, and initiation of EMT. Concurrently, imbalances in the ubiquitination system directly influence the stability of key oncogenes (e.g., Myelocytomatosis oncogene (MYC), β-catenin) and tumor suppressor genes (e.g., p53), thereby enhancing TNBC cell survival and adaptability [26]. Consequently, ubiquitination serves not only as a molecular “trigger” for the early malignant transformation of TNBC but also as a critical supporting mechanism for its progression, drug resistance, and immune evasion, providing a pivotal entry point for understanding the unique malignant biological characteristics of TNBC [27].

Compared with conventional therapeutic targets, the development of ubiquitination-based strategies does not replace existing clinical approaches but rather serves as a complementary modality. These strategies primarily address two critical gaps in TNBC treatment. First, they provide an innovative avenue to target traditionally “undruggable” proteins, allowing key oncogenic factors previously considered intractable to be effectively regulated through degradation and related ubiquitin-mediated mechanisms. Second, they intervene at pivotal nodes associated with drug resistance, including enhanced DNA repair via ubiquitin-specific protease 15/poly (ADP-ribose) polymerase 1 (USP15/PARP1), dynamic modulation of immune checkpoints (e.g., PD-L1 ubiquitination), and ferroptosis resistance. Collectively, these approaches offer promising strategies to overcome resistance to chemotherapy, targeted therapy, and immunotherapy [28]. In this review, we use a stage-specific framework as a pragmatic way to organize the evolving biological and clinical features of the disease across its course. Importantly, the proposed stage-specific framework is intended as an evidence-informed biological organization model rather than a rigid clinicopathological classification system. The framework integrates recurrent molecular characteristics, representative biomarkers, and predominant ubiquitination-associated processes reported across TNBC studies, including stemness maintenance, EMT activation, DNA repair adaptation, ferroptosis resistance, and immune escape. Although partial overlap between stages may occur during tumor evolution, these operational biological contexts provide a useful structure for interpreting the dynamic roles of ubiquitination during TNBC progression. Therefore, this article focuses on the functional network of ubiquitination in TNBC, systematically reviewing its molecular mechanisms in radiotherapy resistance, lipid metabolism reprogramming, and immune microenvironment remodeling. It also addresses potential signaling crosstalk among these processes and further explores possible targeted intervention strategies. By elucidating the central role of post-translational modifications in TNBC progression, this article aims to provide novel theoretical foundations and research directions for overcoming therapeutic challenges in TNBC and developing innovative targeting strategies.

## 2 Stage-Specific Roles of Ubiquitination in TNBC Progression

Although ubiquitin-like modifications share a conserved E1–E2–E3 conjugation logic with ubiquitination, their biological outputs are often distinct. Whereas ubiquitination is widely involved in proteasomal degradation as well as non-degradative signaling, UBLs such as SUMO, NEDD8, and ISG15 more frequently regulate protein localization, interaction networks, complex assembly, and context-specific stress responses. Importantly, these pathways do not act independently. Ubiquitination and UBL modifications can compete for similar lysine residues, occur sequentially on the same substrate, or influence each other through shared enzymes and recognition factors. For instance, SUMOylated proteins can be recognized by SUMO-targeted ubiquitin ligases, linking SUMOylation directly to ubiquitin-mediated downstream processing. Therefore, the functional impact of UBLs should be interpreted within an integrated post-translational modification network rather than as isolated signals. Before examining the specific roles of ubiquitination in TNBC progression, it is essential to systematically evaluate its functional characteristics at different stages of tumor development. As a dynamic and highly adaptable protein modification, ubiquitination exerts multiple functions in tumor initiation, metastatic progression, and therapeutic resistance [29]. To improve biological interpretability, the proposed stage-specific framework is further anchored to representative molecular and phenotypic characteristics recurrently reported in TNBC studies. Rather than defining strict clinicopathological boundaries, each stage is associated with predominant biological behaviors, representative biomarkers, and ubiquitination-associated regulatory networks summarized in Table 1. These operational features are intended to provide a more evidence-informed biological context for distinguishing tumor initiation, progression, and treatment resistance/immune adaptation states within the current literature landscape. Importantly, these molecular features are not proposed as stage-exclusive or clinically validated biomarkers. Rather, they represent recurrent biological characteristics and ubiquitination-associated regulatory patterns more frequently linked to particular functional states during TNBC progression according to currently available mechanistic and translational studies.

**Table 1:** Predominant biological contexts and recurrent ubiquitination-associated features across the proposed TNBC progression framework.

Proposed Stage	Predominant Biological Characteristics	Recurrently Associated Molecular Features	Representative Ubiquitination-Associated Pathways	Primary Evidence Context	Ref.
Tumor initiation stage	Stemness maintenance, genomic instability, DNA damage tolerance, early metabolic adaptation	SOX9 ↑, USP15 ↑, PARP1 stabilization, NF-κB activation, BRCA-associated instability	BCA2-TLR4/MyD88-NF-κB-SOX9 axis; USP15-PARP1 axis	Mainly TNBC-specific mechanistic and preclinical evidence	[30]
Tumor progression stage	EMT activation, invasion, migration, stem cell plasticity, metastatic adaptation	TWIST1 ↑, YAP/TAZ activation, BIRC6 ↑, reduced E-cadherin	USP29-TWIST1; RBX1-FBXO45-TWIST1; USP1-TAZ axis	TNBC-specific *in vitro*/*in vivo* evidence	[31,32]
Treatment resistance and immune escape stage	Ferroptosis resistance, adaptive survival signaling, immune suppression, therapy adaptation	PD-L1 stabilization, GPX4 ↑, SLC7A11 ↑, enhanced DNA repair activity	USP7/USP35-GPX4/SLC7A11; MARCH8-PD-L1; USP22-PD-L1 axis	Mixed translational, preclinical, and clinical association evidence	[33,34,35]

### 2.1 Initial Stage of Tumors: Ubiquitination Networks Predispose Malignant Trends and Initiate Stemness Programs

**Figure 1:**
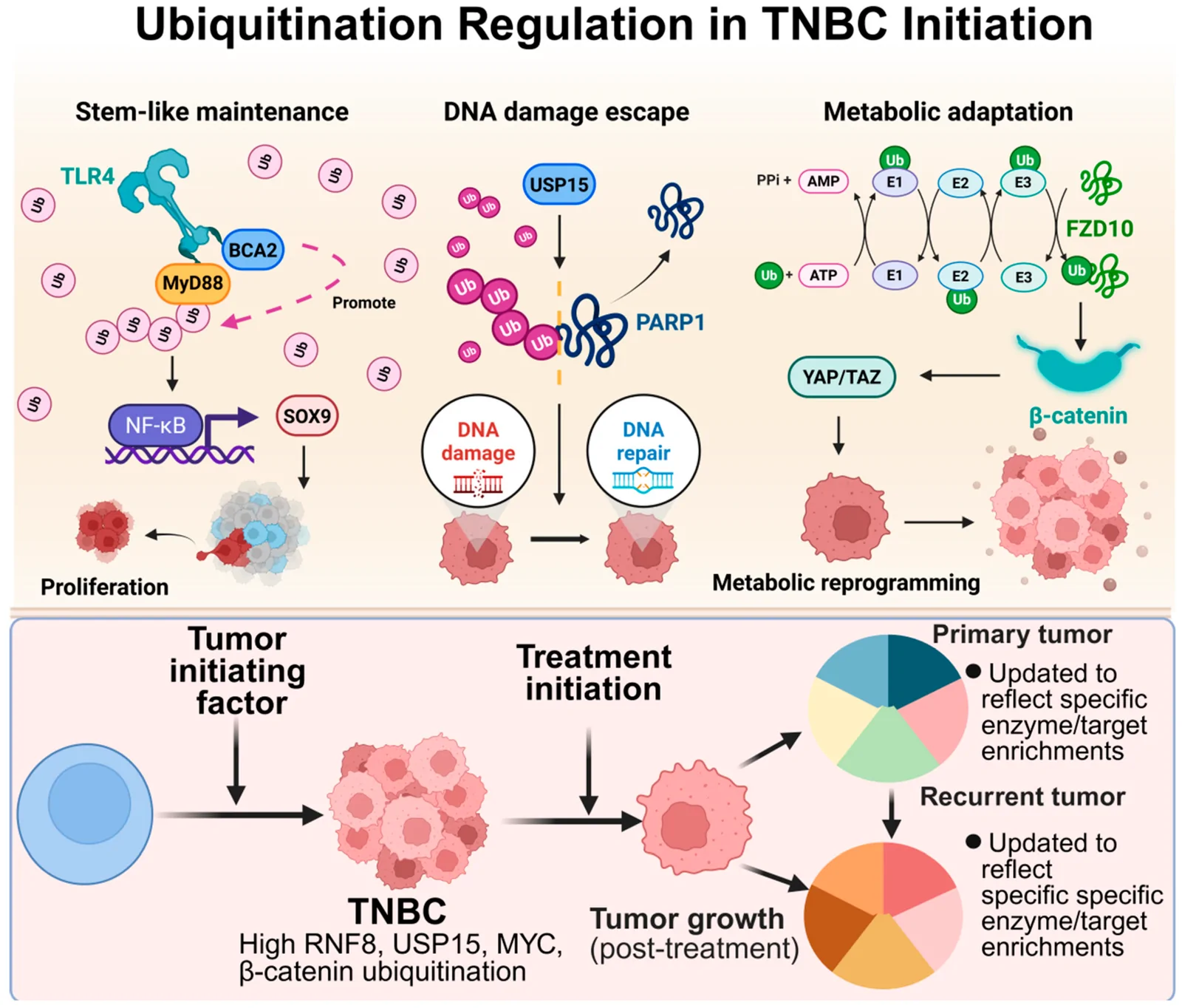
Schematic diagram of the regulatory role of ubiquitination modification in the initial stage of TNBC. Ubiquitination plays a central role in the initiation of TNBC by regulating key axes such as BCA2-TLR4 and USP15-PARP1, presupposing the malignant tendency of the tumor, and initiating stem cell characterization processes. TNBC: Triple-negative breast cancer; TLR4: Toll-like receptor 4; BCA2: Disodium bicinchoninate; MyD88: Myeloid differentiation primary response 88; SOX: SRY-box transcription factor 9; NF-κB: Nuclear factor kappa-B; USP15: Ubiquitin specific peptidase 15; PARP1: Poly ADP-ribose polymerase 1; YAP/TAZ: Yes-associated protein 1/transcriptional coactivator with PDZ-binding motif.

At the tumor initiation stage, the characteristics of the cells are marked by enhanced stem cell properties, genomic instability, and an increased tolerance to DNA damage. The initial stage of tumorigenesis is often accompanied by key biological processes, including stemness maintenance, DNA damage evasion, and metabolic adaptation. Recent studies have increasingly highlighted that ubiquitination plays a central regulatory role in TNBC tumor initiation by targeting diverse signaling proteins and epigenetic regulators (Fig. 1). Compared with other breast cancer subtypes, TNBC is characterized by pronounced genomic instability, stem cell–like properties, and an early capacity to evade programmed cell death, features that are closely associated with its distinctive ubiquitination regulatory network. The E3 ubiquitin ligase BCA2, also known as Ring Finger Protein 115 (RNF115), is highly expressed in breast cancer and has been implicated in maintaining tumor stemness. Notably, Zheng et al. demonstrated that BCA2 inhibits the ubiquitin-mediated degradation of the Toll-like receptor 4/Myeloid Differentiation Primary Response 88 (TLR4/MyD88) complex in TNBC, thereby stabilizing this signaling axis and promoting sustained downstream activation of Nuclear Factor kappa-B (NF-κB) [30]. Activated NF-κB upregulates the stemness marker SRY-box transcription factor 9 (SOX9), maintaining the self-renewal capacity of breast cancer stem cells (BCSCs). This BCA2–TLR4/MyD88–NF-κB–SOX9 axis not only promotes the early formation of a “dormant” tumor cell state but also establishes a foundation for subsequent invasiveness and drug resistance.

Deubiquitinating enzymes (DUBs) also play a crucial role in the early stages of TNBC formation. Sun et al. and colleagues demonstrated that USP15 stabilizes the DNA repair protein PARP1 through deubiquitination, thereby enhancing its activity and improving the capacity of tumor cells to repair DNA damage [2]. This mechanism allows TNBC cells to evade programmed cell death in response to initial genomic instability or chemotherapy-induced stress, providing a molecular basis for subsequent acquired drug resistance and tumor recurrence. These interconnected regulatory events are summarized schematically in Fig. 1 to illustrate how ubiquitination-associated networks contribute to early TNBC malignant transformation and stemness establishment. Notably, ubiquitin specific peptidase 15 (USP15) expression is frequently elevated in TNBC and is positively correlated with poor prognosis following chemotherapy, further underscoring its functional importance in TNBC initiation. In addition, ubiquitination may intersect with epigenetic regulation and contribute to early metabolic reprogramming [2]. For example, the Wnt receptor Frizzled Class Receptor 10 (FZD10) can be ubiquitinated and degraded in TNBC. Wang et al. showed that FZD10 levels in hepatocellular carcinoma (HCC) are regulated by m6A methylation, which in turn modulates the stability of the Hippo pathway core protein Large Tumor Suppressor Kinase 1 (LATS1) [36]. Although this study was conducted using a liver cancer model, the same ubiquitination-m6A-YAP/TAZ regulatory axis may also play a role in TNBC and warrants further investigation. At present, evidence supporting this regulatory axis in TNBC remains indirect, as the available data are derived predominantly from non-TNBC tumor models without direct TNBC-specific *in vitro*, *in vivo*, or clinical validation. Therefore, this pathway should currently be considered exploratory and hypothesis-generating in the TNBC context. FZD10 promotes self-renewal, tumorigenicity, and metastasis of liver cancer stem cells (CSCs) by activating β-catenin and Yes Associated Protein 1 (YAP1), indirectly influencing Yes-associated protein 1/Transcriptional coactivator with PDZ-binding motif (YAP/TAZ)-mediated proliferation and stemness-related transcriptional programs [36]. These findings suggest that crosstalk between m6A and ubiquitination signaling pathways may constitute a core network governing early metabolic adaptation and cell fate determination in TNBC.

High-throughput ubiquitin profiling has further revealed that the expression landscapes of E3 ligases (e.g., Ring Finger Protein 8 (RNF8) [37], Baculoviral IAP Repeat-Containing 6 (BIRC6) [38], HECT, UBA And WWE Domain Containing E3 Ubiquitin Protein Ligase 1 (HUWE1), Tripartite motif containing 25 (TRIM25)) and DUBs (e.g., USP7, USP15) in TNBC distinctively differ from hormone receptor-positive subtypes. Aberrant stability of their substrates—such as MYC, β-catenin, and Notch Receptor 1 (NOTCH1)-suggests that the ubiquitination network “presets” the malignant trajectory of TNBC cells even prior to frank tumor formation. Among these mechanisms, the USP15-PARP1 axis and stemness-associated NF-κB signaling currently possess comparatively stronger support because they have been validated across mechanistic, preclinical, and translational studies and are directly associated with therapeutic resistance phenotypes in TNBC.

### 2.2 Tumor Progression Stage: Ubiquitination Regulates EMT and Invasion–Metastasis Mechanisms

**Figure 2:**
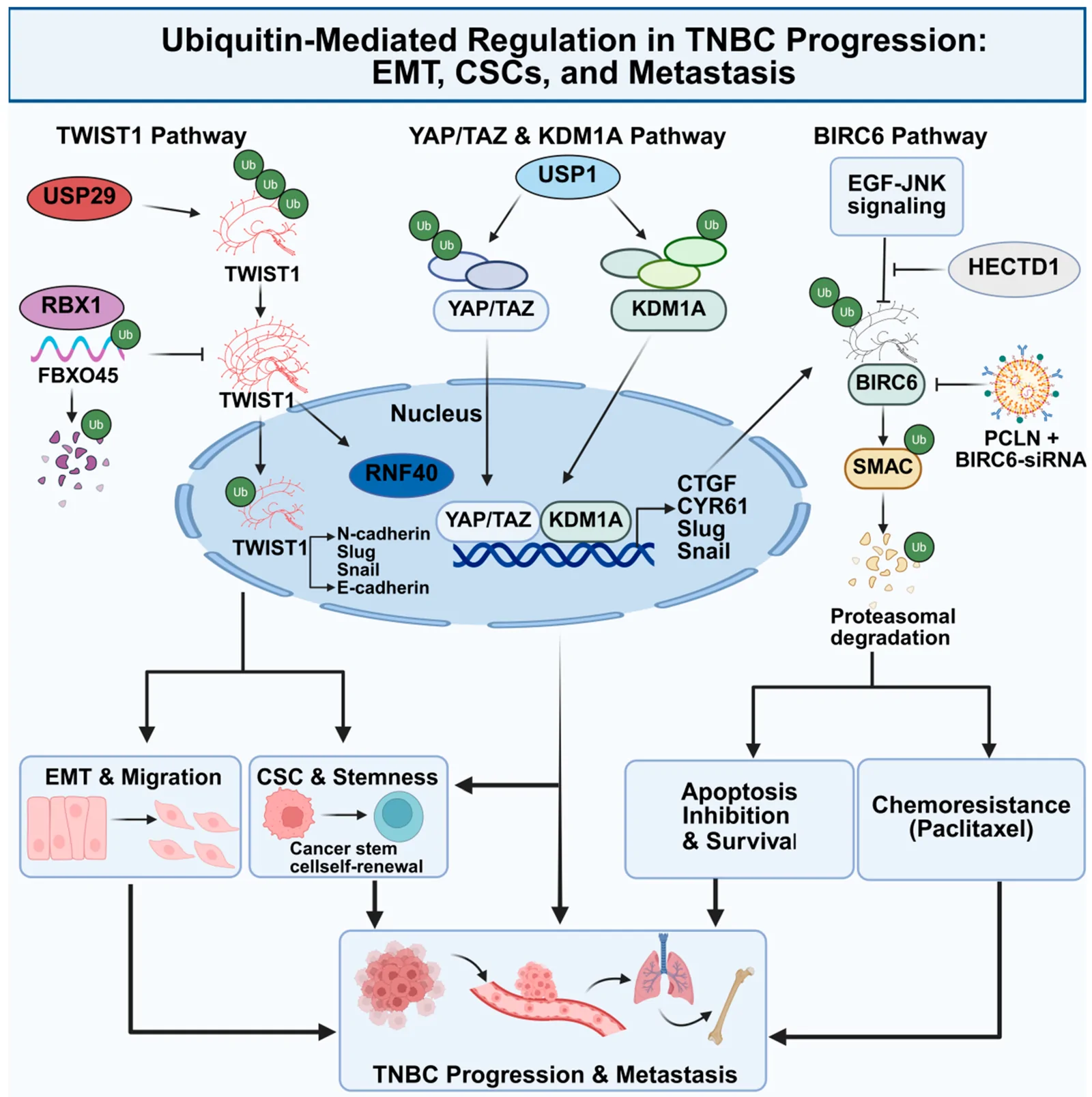
Schematic diagram of the regulatory role of ubiquitination modification in the development stage of TNBC. Ubiquitination modification drives epithelial-mesenchymal transition (EMT) and promotes tumor invasion and metastasis during TNBC progression by regulating pathways such as USP29-TWIST1 and RBX1-FBXO45. TNBC: Triple-Negative Breast Cancer; EMT: Epithelial-Mesenchymal Transition; CSC: Cancer Stem Cell; USP: Ubiquitin Specific Peptidase; TWIST1: Twist Family HLH Transcription Factor 1; KDM1A: Lysine Demethylase 1A; BIRC6: Baculoviral IAP Repeat-Containing 6; EGF/JNK: Epidermal Growth Factor/Jun N-Terminal Kinase; HECTD1: HECT Domain E3 Ubiquitin Protein Ligase 1.

During tumor progression, it is characterized by EMT, increased invasiveness, and enhanced metastatic potential. EMT induces tumor cells to lose epithelial characteristics and acquire migratory and invasive capabilities, accompanied by enhanced stemness, apoptosis resistance, and immune evasion [39]. As a core mechanism for dynamically regulating protein stability and signaling, ubiquitination plays a pivotal role in EMT and metastasis progression in TNBC [40] (Fig. 2). Twist Family BHLH Transcription Factor 1 (TWIST1), a master regulator of epithelial–mesenchymal transition (EMT), plays a pivotal role in cancer stem cell (CSC) self-renewal and metastasis. The stability of TWIST1 is tightly regulated by the ubiquitin–proteasome system. Notably, Guan et al. demonstrated that USP29 directly deubiquitinates and stabilizes TWIST1, a process that is essential for maintaining EMT phenotypes and conferring chemotherapy resistance in TNBC [41]. Complementing this, Shao et al. identified that the E3 ligase RBX1 promotes the ubiquitination-mediated degradation of the EMT inhibitor F-Box Protein 45 (FBXO45). The loss of FBXO45 prevents it from suppressing TWIST1, leading to TWIST1 accumulation and the activation of canonical EMT markers, such as E-cadherin downregulation and N-cadherin upregulation [32]. The RBX1–FBXO45–TWIST1 signaling axis constitutes a non-transcriptional EMT regulatory mechanism, closely associated with enhanced cell migration and invasion. Notably, increasing evidence has highlighted TWIST1 as a critical regulatory hub downstream of factors such as Ring Finger Protein 40 (RNF40) [42], E-cadherin [43], and β-catenin [43], all of which significantly contribute to TNBC-related EMT. Therefore, targeting TWIST1 via ubiquitination modulation may represent a promising therapeutic strategy in TNBC. However, not all EMT-associated ubiquitination pathways appear to contribute equally across TNBC contexts. While several studies identify TWIST1 stabilization as a major driver of metastatic progression, other reports suggest that YAP/TAZ-associated signaling or SNAIL/ZEB1-centered pathways may exert stronger influence in specific mesenchymal-like or basal-like TNBC subtypes. These apparently divergent findings likely reflect differences in molecular subtype composition, experimental models, and treatment-associated selective pressures, supporting the concept that EMT-associated ubiquitination networks in TNBC are dynamically context-dependent rather than regulated through a single universal axis.

Baculoviral IAP Repeat-Containing 6 (BIRC6), an anti-apoptotic protein with E2/E3 dual functions, is often highly expressed in TNBC. A study by Li et al. on the mechanism of TNBC showed that EGF-JNK signaling enhances the stability of BIRC6, preventing its ubiquitination and degradation mediated by the E3 ubiquitin ligase HECTD1 [38]. In turn, BIRC6 reduces SMAC expression by inducing the ubiquitin-proteasome pathway, thereby antagonizing apoptosis and promoting the proliferation, colony formation, tumor sphere formation, and growth ability of TNBC cells [38]. As a result, while TNBC cells acquire migratory properties, they also exhibit resistance to endogenous apoptotic signals, improving their ability to survive in circulation and in distant organs. BIRC6 not only promotes metastasis but is also associated with resistance to paclitaxel, suggesting that it serves as a dual-functional node for both metastasis and drug resistance. Additionally, PEGylated cationic lipid nanoparticles (pCLN) in an LNP system efficiently deliver BIRC6-siRNA into TNBC cells both *in vitro* and *in vivo*. siRNA significantly downregulates BIRC6 expression in TNBC, inhibiting cell growth and tumorigenesis without adverse effects [44].

Furthermore, the Hippo pathway effectors YAP/TAZ are central to cell density-dependent proliferation and invasion. In TNBC, USP1 has been shown to stabilize both KDM1A [45] and TAZ [46]. Specifically, USP1 removes K48-linked ubiquitin chains from TAZ, preventing its degradation and promoting its nuclear accumulation. This drives the transcription of downstream targets like connective tissue growth factor (CTGF) and Cysteine rich 61 (CYR61), reinforcing EMT and stemness programs. Consequently, disrupting these specific deubiquitination events offers a strategy to mitigate the high metastatic potential of TNBC [47,48]. As illustrated in Fig. 2, these EMT-associated ubiquitination pathways collectively contribute to invasion, migration, stemness maintenance, and metastatic adaptation during TNBC progression.

### 2.3 Treatment Resistance and Immune Escape: The Dual Regulatory Role of Ubiquitination

**Figure 3:**
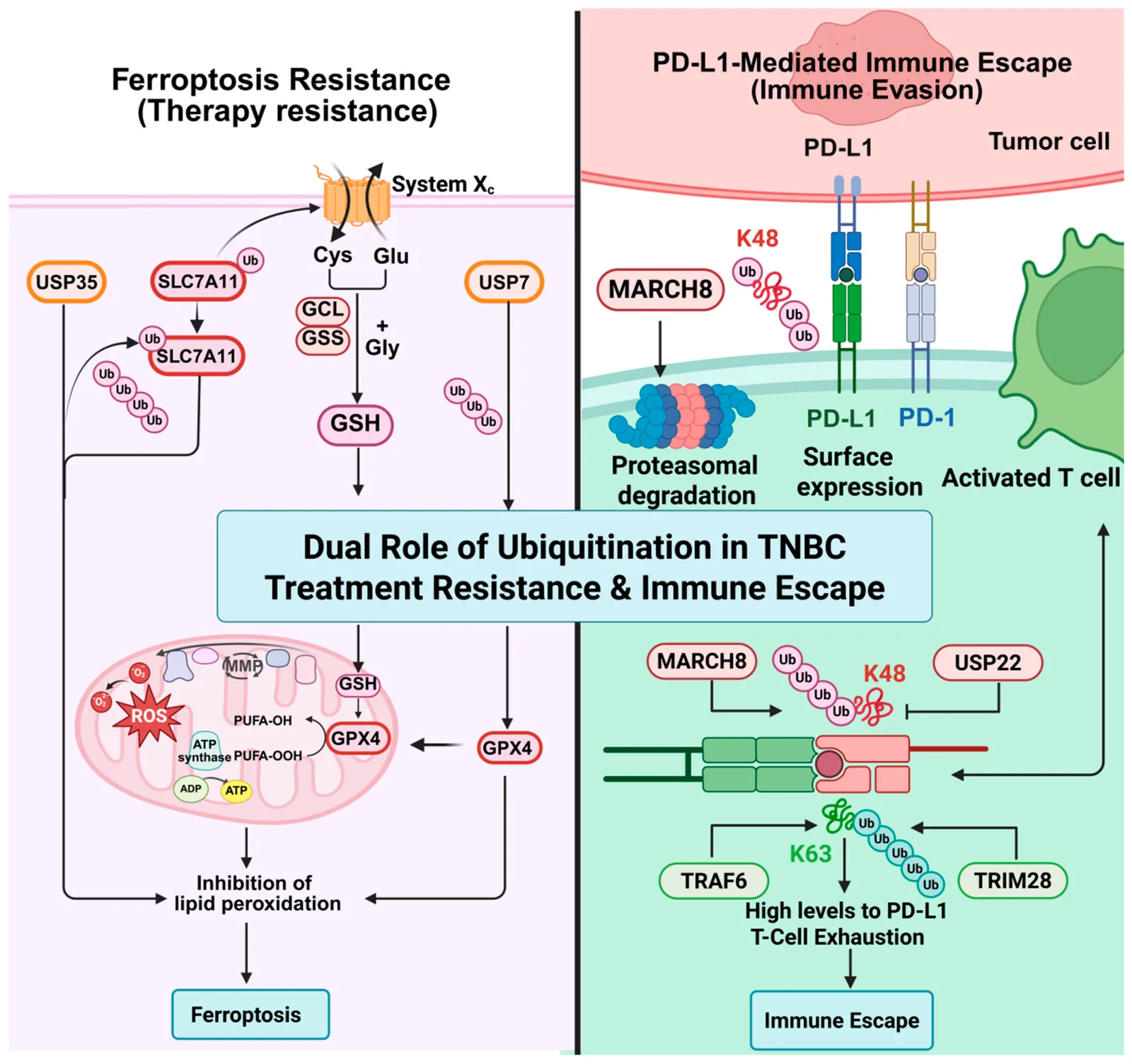
Schematic diagram of the regulatory role of ubiquitination modification in the initial drug resistance stage of TNBC. Ubiquitination plays a dual role in TNBC treatment resistance and immune evasion, including inhibiting ferroptosis by stabilizing GPX4/SLC7A11 and dynamically regulating PD-L1 stability to evade immune attack. TNBC: Triple-Negative Breast Cancer; GPX4: Glutathione Peroxidase 4; TRAF6: TNF Receptor Associated Factor 6; SLC7A11: Solute Carrier Family 7 Member 11; PD-L1: Programmed Cell Death Ligand 1; MARCH8: Membrane-Associated RING-CH 8; USP22: Ubiquitin Specific Peptidase 22; TRIM28: Tripartite Motif Containing 28.

The clinical management of TNBC is severely challenged by acquired resistance to chemo-/radiotherapy and immune evasion [49] (Fig. 3). During the treatment resistance and immune escape stage, it is associated with ferroptosis resistance, activation of immune checkpoints, and the activation of adaptive survival mechanisms in response to treatment stress. Ubiquitination governs these processes by regulating cell death pathways and immune checkpoint dynamics [50]. Ferroptosis, an iron-dependent form of regulated cell death, has emerged as a key vulnerability in TNBC. However, tumor cell often develop resistance by manipulating the stability of ferroptosis suppressors via Deubiquitinating enzymes (DUBs) [51]. For example, USP7 and USP35 selectively remove K48-linked ubiquitin chains from Glutathione Peroxidase 4 (GPX4) and Solute Carrier Family 7 Member 11 (SLC7A11), thereby preventing their proteasomal degradation. This stabilization enhances the cellular capacity to detoxify lipid peroxides, ultimately conferring resistance to ferroptosis-inducing agents [52,53]. Importantly, inhibiting ferroptosis limits their release of damage-associated molecular patterns (DAMPs), thereby impairing dendritic cell activation and T-cell recruitment, creating an immunosuppressive microenvironment [54,55].

Programmed death-ligand 1 (PD-L1), a key immune checkpoint molecule, plays a central role in tumor immune evasion, with its expression directly affecting T cell activity and clinical responses to immunotherapy. Recent studies have revealed that PD-L1 protein stability is dynamically regulated by diverse ubiquitination events, with specific ubiquitin chain linkages critically determining its degradation and functional maintenance [56,57]. In TNBC, K48-type ubiquitination, typically mediated by the E3 ubiquitin ligase MARCH8, promotes PD-L1 proteasomal degradation, thereby attenuating its immunosuppressive function. In contrast, K63-type ubiquitination, mediated by E3 ligases such as TRAF6 [58] and TRIM28 [59], stabilizes PD-L1 on the cell membrane and enhances its downstream signaling, promoting immune evasion. Recent evidence suggests that USP22 contributes to tumor immune evasion by sustaining PD-L1 expression through deubiquitination-dependent stabilization mechanisms. In DLBCL cells, USP22 was reported to stabilize β-catenin and facilitate its nuclear localization, thereby promoting PD-L1 expression and suppressing CD8^+^ T-cell activity [60]. This reversible and dynamic ubiquitination–deubiquitination process enables TNBC cells to finely tune PD-L1 expression according to the microenvironment, balancing immune surveillance and T cell exhaustion to ensure long-term tumor survival [61]. Clinical evidence further underscores the functional relevance of this mechanism: high USP22 expression is closely associated with increased PD-L1 protein levels and significantly correlates with poor responses to immune checkpoint inhibitor (ICI) therapy [62]. Importantly, the mechanistic evidence summarized below varies in strength, ranging from TNBC-specific functional studies to broader preclinical or cross-cancer observations. Therefore, the translational significance of individual pathways should be interpreted according to their current level of experimental and clinical support. Therefore, targeted regulation of PD-L1 ubiquitination/deubiquitination is emerging as a research hotspot (Table 2). Inhibiting DUBs that enhance PD-L1 stability or blocking K63-type ubiquitination-dependent signaling pathways may reduce TNBC immune evasion and improve immunotherapy efficacy [63]. This strategy not only deepens understanding of the PD-L1 regulatory network but also provides a potential avenue for developing novel combination treatment approaches [64]. Fig. 3 integrates the major ubiquitination-associated mechanisms involved in treatment resistance and immune escape, highlighting the functional interactions among ferroptosis regulation, PD-L1 stabilization, and adaptive survival signaling. Notably, PD-L1-associated ubiquitination pathways currently possess comparatively stronger translational relevance because they are directly linked to clinically implemented immune checkpoint blockade strategies, including PD-1/PD-L1-targeted therapies evaluated in TNBC clinical trials such as IMpassion130 and KEYNOTE-355. By contrast, several ferroptosis-associated mechanisms, although mechanistically compelling, remain predominantly supported by preclinical observations and have not yet achieved comparable levels of clinical validation in TNBC.

To improve evidence transparency and interpretability, the major ubiquitination-associated pathways discussed in this review were stratified according to TNBC-specific *in vitro* evidence, *in vivo* validation, patient-associated findings, cross-cancer extrapolated observations, and current translational status, as summarized in Table 2.

**Table 2:** Regulatory Effects of Ubiquitination on Key TNBC Proteins.

Regulator/Pathway	TNBC *In Vitro* Evidence	TNBC *In Vivo* Evidence	Patient/Clinical Association	Cross-Cancer Extrapolated Evidence	Principal Biological Relevance	Translational Status	Ref.
BCA2-TLR4/MyD88-NF-κB-SOX9	Yes	Limited	Limited	No	Stemness maintenance and tumor initiation	Exploratory	[30]
USP15-PARP1	Yes	Yes	Yes	No	DNA repair adaptation and therapeutic resistance	Emerging	[2]
FZD10-YTHDF2-YAP/TAZ	No direct TNBC validation	No	No	Yes (hepatocellular carcinoma)	Stemness and metabolic adaptation	Exploratory	[36]
USP29-TWIST1	Yes	Yes	Limited	No	EMT and metastatic progression	Exploratory	[41]
RBX1-FBXO45-TWIST1	Yes	Limited	No	No	EMT and invasion	Exploratory	[32]
BIRC6	Yes	Yes	Limited	Partial	Apoptosis suppression and metastasis	Emerging	[38]
USP1-TAZ/KDM1A	Yes	Yes	Partial translational association	No	EMT, stemness, and resistance	Translationally supported	[45]
USP7/USP35-GPX4/SLC7A11	Yes	Partial	No direct clinical validation	Partial	Ferroptosis resistance	Emerging	[53]
MARCH8-PD-L1	Yes	Limited	Limited	Partial	Immune escape regulation	Emerging	[35]
TRAF6-PD-L1	Limited TNBC evidence	No	No	Yes	Immune checkpoint regulation	Exploratory	[58]
TRIM28-TBK1/IRF1-PD-L1	Limited TNBC evidence	No	No	Yes	PD-L1 transcriptional activation	Exploratory	[59]

Note: BCA2: Breast Cancer-Associated Gene 2; RNF115: Ring Finger Protein 115; FZD10: Frizzled Class Receptor 10; RBX1: Regulator of Cullins 1; FBXO45: FBXO Protein 45; BIRC6: Baculoviral IAP Repeat-Containing 6; MARCH8: Membrane-Associated RING-CH 8; TRAF6: TNF Receptor Associated Factor 6; PD-L1: Programmed Cell Death Ligand; USP: Ubiquitin-Specific Proteases; TRAF6: TNF Receptor Associated Factor 6; TRIM28: Tripartite Motif Containing 28.

Ubiquitination is well recognized for its central role in proteasome-dependent degradation pathways and is often described as an intracellular “destruction tag” that dictates the fate and abundance of numerous proteins [65]. In addition to ubiquitination, cells utilize a related system of ubiquitin-like modifications, which, although biochemically similar, perform distinct functional roles. The primary distinction lies in their core biological functions: while ubiquitination mainly drives protein degradation and dynamic turnover, serving as a key mechanism for maintaining protein homeostasis, ubiquitin-like modifications act as finely tuned “molecular switches.” These modifications modulate signal transduction networks and cell fate decisions by regulating substrate protein conformation, localization, interactions, and activity [66,67]. Thus, ubiquitination is not merely a “tag for destruction” but constitutes a sophisticated regulatory mechanism [68]. Some regulatory relationships have been established as direct causal links through experiments such as loss-of-function studies, mutation of ubiquitination sites, and *in vitro* ubiquitination assays, while others are primarily based on expression correlations or indirect evidence and require further validation.

## 3 Stage-Specific Role of Ubiquitin-Like (UBL) Modifications in TNBC Progression

**Figure 4:**
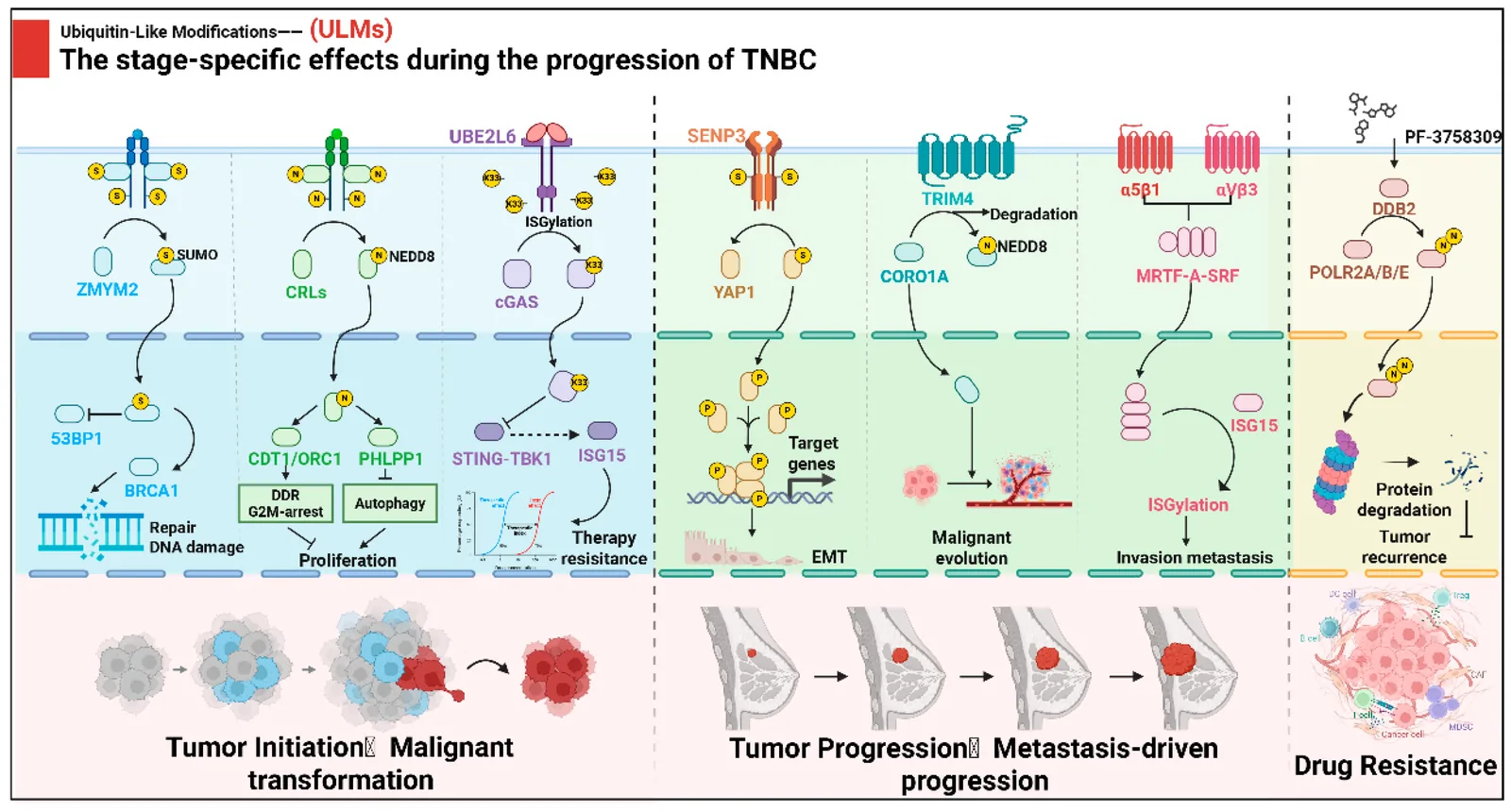
Schematic diagram of the stage-specific role of ubiquitination-like modification in the progression of TNBC. The specific functions of ubiquitin-like modifications such as SUMOylation and NEDD8ylation in different stages of TNBC progression (initiation, development, and resistance) synergistically drive the malignant evolution of tumors through ubiquitination. TNBC: Triple-negative breast cancer; SUMO: Small ubiquitin-like modifier; ZMYM2: Zinc finger MYM-type containing 2; NEDD8: Neural precursor cell-expressed developmentally downregulated 8; ISG15: Interferon-stimulated gene 15 ubiquitin-like modifier; cGAS: Cyclic GMP-AMP synthase; STING: Stimulator of interferon genes; TBK1: TANK binding kinase 1; YAP1: Yes1 associated transcriptional regulator; CORO1A: Coronin 1A; DDB2: Damage-specific DNA-binding protein 2; Tregs: Regulatory T cells; MDSCs: Myeloid-derived suppressor cells; CAFs: Cancer-associated fibroblasts.

UBL modifications are mediated by a family of structurally homologous yet functionally distinct proteins, including SUMO, NEDD8, and ISG15. Similar to ubiquitination, these modifiers are covalently conjugated to substrate proteins through sequential enzymatic cascades involving E1 activating, E2 conjugating, and E3 ligase enzymes (Fig. 4). In tumor biology, and particularly in TNBC, UBL modifications are increasingly recognized as critical regulators of disease initiation, progression, and therapeutic resistance.

### 3.1 Tumor Initiation Stage: UBL Modifications Mediate Genome Instability and Immune Evasion

During the initial phase of TNBC development, UBL modifications are intricately involved in malignant transformation through multiple mechanisms. For instance, SUMOylation plays a central role in the DNA damage response and the maintenance of genomic stability. It facilitates the recruitment and assembly of key DNA repair factors such as BRCA1 and 53BP1, thereby ensuring chromosomal integrity [69]. Dysregulation of SUMOylation, however, can compromise DNA repair efficiency, exacerbating genomic instability—a key driver of TNBC initiation. Similarly, NEDD8ylation regulates the turnover of critical cell cycle regulators—including CDT1, ORC1, p21, p27, and PHLPP1—through the activation of Cullin-RING E3 ubiquitin ligase complexes (CRLs) [70]. While this process normally maintains cell cycle homeostasis, its aberrant activation during early tumorigenesis can confer a sustained proliferative advantage, accelerating the transition from normal epithelium to a malignant phenotype. Furthermore, ISGylation, a UBL modification primarily associated with antiviral and inflammatory responses, can be dysregulated in early tumors. As demonstrated by Peng et al., aberrant ISG15 modification impairs innate immune surveillance and reduces cellular stress clearance, thereby promoting the survival and accumulation of pre-malignant cells [71]. Collectively, UBL modifications not only provide a transformative advantage by regulating DNA repair and cell cycle progression but also foster a permissive microenvironment for tumor onset by modulating immune responses, underscoring their pivotal role in the molecular landscape of TNBC initiation.

### 3.2 Tumor Progression Stage: Multi-Dimensional Driving and Malignant Evolution of Ubiquitination

During the tumor progression stage of TNBC, UBL modifications, similar to ubiquitination, drive invasion, metastasis, and stemness maintenance of tumor cells through multi-level regulatory mechanisms. SUMOylation, for instance, plays a pivotal role in transcriptional regulation and epigenetic modification. It promotes EMT and inflammatory signaling by modulating the activity of transcription factors such as HIF-1α, STAT3, and NF-κB, thereby enhancing the migratory and invasive capacities of TNBC cells. Chen et al. reported that YAP1 is subject to SUMOylation, and its deSUMOylation by SENP3 promotes degradation via the ubiquitin–proteasome system, consequently suppressing migration, invasion, and stem cell-like phenotypes in TNBC [72]. These findings suggest that SUMOylation and ubiquitination function coordinately on YAP1, where SUMOylation is associated with YAP1 stabilization, while SENP3-mediated deSUMOylation facilitates its subsequent degradation through the ubiquitin–proteasome pathway. Likewise, NEDD8 modification regulates several oncogenic pathways, including TGF-β and Wnt/β-catenin, through CRL activation. These pathways are critical for maintaining cancer stemness, hypoxia adaptation, and angiogenesis, thereby enhancing the survival and proliferative potential of TNBC cells. Related studies have emphasized the therapeutic potential of targeting pathogenic proteins through the NEDD8 pathway [73]. Additionally, ISGylation exhibits unique functions during tumor progression. It reshapes cell migration characteristics by modifying cytoskeletal and adhesion proteins and may suppress anti-tumor immune responses, enabling tumor cells to evade immune clearance. Hermann et al. found that the malignant breast cancer cell line MDA-MB-231 expressed high levels of β1 integrin, ISG15, and ISGylated proteins, which collectively promoted invasiveness [74]. Collectively, UBL modifications not only enhance the invasiveness and migration of TNBC cells during progression but also establish a molecular basis for tumor dissemination and heterogeneity by maintaining stemness and modulating the immune microenvironment, highlighting their potential as therapeutic targets.

### 3.3 Tumor Drug Resistance and Recurrence Stage: UBL Modifications Mediate DNA Repair Barriers and Treatment Resistance

During the phases of acquired resistance and relapse, UBL modifications significantly contribute to treatment failure. SUMOylation activity within DNA repair pathways is closely associated with chemotherapy resistance. Excessive SUMOylation has been shown to enhance homologous recombination and nucleotide excision repair efficiency, thereby diminishing the efficacy of DNA-damaging agents such as cisplatin and PARP inhibitors, allowing residual tumor cells to survive and ultimately leading to disease recurrence [75,76]. Similarly, NEDD8 modification contributes to the development of drug-resistant phenotypes by regulating DNA damage repair. For instance, Jia et al. reported that NEDD8 participates in both DNA repair and protein degradation pathways. Specifically, the E3 ubiquitin ligase DDB2 facilitates the ubiquitin-dependent degradation of POLR2A, POLR2B, and POLR2E via the cullin-RING ligase (CRL) pathway [77]. This enhanced protein turnover not only facilitates tumor cell survival under drug-induced DNA damage but also increases adaptability to targeted therapies, ultimately contributing to clinical treatment failure and tumor recurrence. Collectively, UBL modifications establish multiple protective barriers for tumor cells by enhancing DNA repair, maintaining survival signaling, and modulating immune responses during the drug resistance and relapse stages of TNBC [78,79]. These characteristics reveal the molecular basis of refractory recurrence in TNBC and suggest that targeted interventions on UBL modifications may represent a promising strategy to overcome drug resistance and improve patient prognosis [80].

In general, UBL modifications exert stage-specific regulatory effects throughout TNBC progression (Table 3). During tumor initiation, they influence malignant transformation by modulating DNA repair, cell cycle progression, and immune surveillance. In the progression phase, they drive proliferation by promoting EMT, maintaining stemness, and reshaping the immune microenvironment. During resistance and relapse, they enhance treatment failure by improving DNA repair capacity, sustaining survival pathways, and facilitating immune escape [72]. A deeper understanding of these stage-specific roles will not only elucidate the molecular complexity of TNBC but also provide valuable insights for the development of temporally precise therapeutic strategies. These stage-associated functions of UBL modifications are further integrated in Fig. 4, which summarizes their coordinated roles in TNBC initiation, progression, resistance, and immune adaptation.

**Table 3:** Functional Regulation of Key Proteins in TNBC by Ubiquitin-Like Modifications.

Disease Stage	UBL Modification	Key Target/Pathway	Principal Biological Function	Evidence Category	Ref.
Tumor initiation	SUMOylation	BRCA1/53BP1	DNA repair regulation and genome stability maintenance	Broad cancer mechanistic evidence with TNBC relevance	[69]
Tumor initiation	SUMOylation	YAP1	Regulation of tumor initiation and stemness-associated signaling	TNBC-specific mechanistic evidence	[72]
Tumor initiation	NEDDylation	CRLs	Cell-cycle regulation and DNA damage response	Broad cancer preclinical evidence	[70]
Tumor initiation	ISGylation	UBE2L6	Immune suppression and innate immune modulation	Cross-cancer mechanistic evidence	[71]
Tumor progression	SUMOylation	YAP1	EMT, migration, invasion, and stemness regulation	TNBC-specific mechanistic evidence	[72]
Tumor progression	NEDDylation	CORO1A	Suppression of TNBC progression through targeted degradation	TNBC-specific preclinical evidence	[81]
Tumor progression	ISGylation	ISG15	Promotion of migration and invasion	Breast cancer-associated mechanistic evidence	[74]
Treatment resistance	SUMOylation	BRCA1/53BP1	Enhanced DNA repair and chemoresistance	Broad cancer translational evidence	[69]
Treatment resistance	NEDDylation	POLR2A/B/E	DNA repair adaptation and treatment resistance	Primarily preclinical evidence	[77]

Note: SUMO: Small ubiquitin-related modifier; BRCA1: Breast cancer susceptibility gene 1; 53BP1: Tumor protein p53 binding protein 1; YAP1: Yes1 associated transcriptional regulator; CRLs: Cullin-RING Ligases; UBE2L6: Ubiquitin-conjugating enzyme E2 L6; NEDD8: Neural precursor cell-expressed developmentally downregulated 8; CORO1A: Coronin 1A; ISG: Interferon-stimulated genes; BRCA1: Breast cancer susceptibility gene 1; 53BP1: Tumor protein p53 binding protein 1.

The relevance of ubiquitination and UBL modification mechanisms to TNBC is currently biologically plausible but not yet firmly established. Direct evidence in TNBC-specific models remains limited. Moreover, the available support is mainly restricted to preclinical settings, with little or no TNBC-specific clinical validation reported to date.

## 4 Clinical Translation and Recent Advances in TNBC Treatment

As the understanding of ubiquitination and ubiquitin-like (UBL) modification mechanisms in TNBC continues to evolve, increasing attention has shifted from mechanistic insights toward their clinical translation. Importantly, these post-translational modifications not only regulate key oncogenic pathways but also directly influence therapeutic sensitivity, resistance, and patient stratification. Therefore, elucidating ubiquitination-dependent regulatory networks provides a mechanistic foundation for optimizing current treatments and developing novel therapeutic strategies. Current translational efforts can be broadly categorized into four interrelated areas: targeted therapy development, rational combination strategies, drug delivery optimization, and biomarker-driven patient selection. Notably, the integration of ubiquitination biology into these domains enables a more precise linkage between molecular mechanisms and clinical decision-making, thereby accelerating the implementation of precision medicine in TNBC [82].

### 4.1 Clinical Applications and Challenges of Immune Checkpoint Inhibitors (ICIs)

Immunotherapy now stands at the forefront of TNBC research. Anti-PD-1/PD-L1 monoclonal antibodies, including atezolizumab and pembrolizumab, have shown potential to improve progression-free survival (PFS) and overall survival (OS) in PD-L1-positive TNBC, as demonstrated in trials such as IMpassion130 and KEYNOTE-355 [83]. In the IMpassion130 trial, the addition of atezolizumab to nab-paclitaxel extended median progression-free survival (PFS) from 5.0 to 7.5 months in PD-L1–positive patients, highlighting the potential benefit of chemoimmunotherapy combinations in selected populations. However, no overall survival (OS) benefit was observed in the entire cohort, and these findings were not replicated in IMpassion131, emphasizing the critical importance of patient selection and optimized combination strategies [84]. The conflicting outcomes of IMpassion130 and IMpassion131 are likely multifactorial, possibly reflecting differences in chemotherapy regimens, corticosteroid premedication, and trial population characteristics. Importantly, these findings suggest that the efficacy of ICIs in TNBC may depend on the specific chemotherapy partner rather than representing a uniform class effect. Similarly, KEYNOTE-355 reported significant PFS and OS improvement with pembrolizumab plus chemotherapy only in patients with PD-L1 CPS ≥ 10, highlighting the role of predictive biomarkers. Despite these advances, the overall objective response rate remains below 30%, and resistance remains common [85]. Notably, PD-L1 protein stability is dynamically regulated by ubiquitination and deubiquitination, directly influencing immunotherapy responses. For instance, the E3 ubiquitin ligase MARCH8 mediates K48-linked ubiquitination and proteasomal degradation of PD-L1, thereby enhancing T cell activity. In contrast, deubiquitinating enzymes such as USP22 and CSN5 remove K48-linked chains to stabilize PD-L1, prolong its half-life, and facilitate immune evasion. Clinically, elevated USP22 expression is associated with poor responses to immune checkpoint inhibitors (ICIs), highlighting that therapeutic modulation of PD-L1 ubiquitination may improve clinical outcomes [86]. Further translational studies reveal that multi-layered immunosuppression within the tumor microenvironment (TME) constrains immunotherapy efficacy. Ubiquitin-like modifications, including ISGylation, can further dampen anti-tumor immunity by blunting innate immune responses and disrupting interferon signaling. Additionally, the high molecular heterogeneity and low neoantigen stability in TNBC reduce immunogenicity, contributing to the variable response to ICIs [87]. To overcome these limitations, current clinical investigations are exploring combination strategies aimed at boosting response rates. These include integrating immunotherapy with metabolic modulators, epigenetic agents, or novel immunomodulators. For instance, MCT1 inhibitors lower lactate accumulation in tumors and improve immune cell function, while small-molecule inhibitors targeting ubiquitination-related enzymes (e.g., USP22, CSN5) or key UBL-modifying enzymes (e.g., UBE2L6) may synergize with ICIs by reversing the immunosuppressive TME [88].

Advances in multi-omics and immune profiling are facilitating the precise identification of TNBC patients likely to benefit from immunotherapy, shifting its application from a “partially sensitive” approach toward a more personalized and widely applicable strategy. Concurrently, integrative analyses of UBL modifications are poised to reveal novel predictive biomarkers and druggable targets, ultimately enhancing the clinical efficacy of immunotherapy in TNBC. Collectively, these findings underscore that ubiquitination-mediated regulation of PD-L1 is not merely a molecular event but represents a clinically actionable mechanism that can be leveraged to improve immunotherapy outcomes and overcome therapeutic resistance in TNBC.

### 4.2 PARP Inhibitors and Precision Treatment of DNA Repair Defects

While poly(ADP-ribose) polymerase inhibitors (PARPi) show promise in homologous recombination deficiency (HRD) TNBC, their clinical efficacy is still limited, underscoring the need for novel targets to enhance treatment response [89]. Agents like olaparib and talazoparib are approved for germline BRCA1/2-mutant TNBC, marking progress in precision therapy; however, their effectiveness remains suboptimal in BRCA wild-type patients [90]. Recent translational studies point to compensatory activation of DNA repair as a key resistance mechanism. Notably, ubiquitination dynamically regulates core DNA-repair proteins: the ubiquitination status of RAD51 and BRCA1, for instance, critically governs their stability and recruitment, thereby modulating cellular sensitivity to PARPi [91]. Targeting specific E3 ligases (e.g., RNF8, RNF168) or deubiquitinating enzymes (e.g., USP1, USP7) can impair homologous recombination-mediated repair and thus potentiate PARPi efficacy [92,93]. This regulatory axis is especially active in the highly glycolytic TNBC tumor microenvironment, offering a metabolic-epigenetic perspective on PARPi resistance. These findings suggest that targeting ubiquitination-dependent DNA repair regulation may serve as a promising strategy to sensitize TNBC tumors to PARP inhibitors, particularly in BRCA-proficient patients who currently derive limited benefit.

### 4.3 Emerging Prospects of Antibody–Drug Conjugates (ADCs)

Antibody-drug conjugates (ADCs) offer a novel therapeutic avenue for TNBC. In the phase III ASCENT trial, the Trop-2-targeting ADC sacituzumab govitecan (SG) significantly improved progression-free survival (PFS) and overall survival (OS) in patients with pretreated metastatic TNBC, leading to its landmark FDA approval for this indication [94]. This approval highlights the potential of precision delivery strategies to overcome chemotherapy resistance. Beyond SG, next-generation ADCs are advancing clinically. Datopotamab deruxtecan (targeting TROP2) [95] and ladiratuzumab vedotin (targeting LIV-1) [96] have shown promising objective response rates and manageable safety in early trials, with some responses outlasting those achieved with conventional chemotherapy.

Recent studies suggest that ubiquitination and ubiquitination-like modifications may play a critical role in ADC resistance and sensitivity. For instance, some cytotoxic drugs (such as topoisomerase I inhibitors) released by ADCs exert their effects by inducing DNA damage [97]. Ubiquitination is central to the dynamic regulation of DNA damage repair proteins, and this process could impact the efficacy of related targeted therapies. These observations raise the possibility that modulation of ubiquitination pathways could influence ADC efficacy by altering DNA damage responses, thereby providing a potential strategy to overcome resistance and improve therapeutic outcomes.

### 4.4 Combined Treatment and Individualized Strategies

TNBC is characterized by significant histopathological, transcriptomic, and genomic heterogeneity, encompassing multiple distinct entities rather than a uniform disease. Therefore, subclassifying TNBC and defining its molecular subtypes are crucial for deciphering its complexity and for advancing targeted, precision medicine strategies [98]. Within this framework, multi-omics analyses of ubiquitination and ubiquitin-like modifications are emerging as sources of novel biomarkers for personalized therapy. Ubiquitination profiling via mass spectrometry-based ubiquitinomics can delineate TNBC-specific activity patterns of E3 ligases and DUBs [22]. Such profiles can be correlated with patient outcomes and drug sensitivity; for instance, high expression of RNF8 and USP15 is often associated with poor responses to DNA-damaging agents, suggesting a potential benefit from early combination with targeted inhibitors. Furthermore, elevated ubiquitination levels are linked to an immunosuppressive tumor microenvironment, which may limit the efficacy of ICIs as monotherapy. Additionally, mutations in genes involved in post-translational modifications—such as amplifications or point mutations in USP15 and USP7—have been closely associated with resistance to various targeted and immunotherapies [99]. Incorporating these genetic alterations into future clinical testing panels could enable more stratified selection of targeted agents and immunotherapies for TNBC patients. Importantly, integrating ubiquitination-related molecular alterations into clinical decision-making frameworks may facilitate more precise patient stratification and guide the selection of targeted therapies and combination regimens. Nevertheless, whether ubiquitination-associated molecular signatures can reliably improve treatment selection or predict long-term therapeutic benefit in TNBC still requires prospective clinical validation. Integration of ubiquitinomics with transcriptomic and clinical response data may help establish more robust precision-stratification strategies in the future.

Overall, bridging ubiquitination-driven molecular mechanisms with therapeutic strategies provides a critical opportunity to translate basic discoveries into clinically actionable interventions in TNBC. Despite compelling mechanistic evidence, the clinical translation of these findings remains incomplete (Table 4). Current evidence from early-phase and exploratory clinical studies suggests that therapeutic modulation of these pathways may offer benefit in selected settings; however, the magnitude and durability of clinical responses have been variable. Importantly, not all mechanistically rational interventions have resulted in meaningful patient benefit, and several approaches have shown limited efficacy, emerging resistance, or unacceptable toxicity in clinical testing. These discrepancies highlight that biological plausibility alone is insufficient for successful translation and that a stronger integration of mechanistic insight with clinical outcome data is required.

**Table 4:** Clinical trial outcomes, linkage between mechanisms and therapeutic impact.

Target/Strategy	Representative Agent	Trial/Clinical Evidence	Reported Outcome or Limitation	TNBC Relevance	Stratification Implication	Ref.
MDM2-p53 axis	AMG-232/KRT-232	Phase I, TP53-wild-type advanced solid tumors/MM, NCT01723020	No objective responses by local review; 3 unconfirmed PRs by central review; SD in some patients; DLTs included thrombocytopenia and neutropenia	Not TNBC-specific; breast cancer cohort showed only short SD	Most relevant to TP53-wild-type/MDM2-amplified tumors, but many TNBCs harbor TP53 mutation	[100,101,102]
USP1 inhibition	KSQ-4279/RO7623066	Phase I, HRR-mutated tumors, NCT05240898	ASCO 2024 reported acceptable safety; MTD not reached; preliminary activity limited, with no mature TNBC-specific efficacy data	Mechanistically relevant to HRR-deficient/BRCA-like TNBC and PARPi sensitization	HRR mutation, BRCA-like phenotype, replication-stress signature	[45,103]
NEDDylation inhibition	Pevonedistat/MLN4924	Phase Ib with docetaxel, carboplatin/paclitaxel, or gemcitabine, NCT01862328	ORR 16 percent with docetaxel and 35 percent with carboplatin/paclitaxel; gemcitabine arm discontinued due to poor tolerability; DLTs included liver enzyme elevation, febrile neutropenia, thrombocytopenia	Not TNBC-specific but relevant because carboplatin/paclitaxel is clinically used in TNBC	ERCC1-high tumors showed longer treatment duration in exploratory analysis	[81,104]

### 4.5 Emerging Technologies Accelerating Discovery

Recent technological advances have substantially expanded the toolkit available for investigating this field. First, ubiquitinomics has provided a systems-level approach for mapping ubiquitinated proteins, identifying modification sites, and characterizing dynamic ubiquitin signaling under physiological and pathological conditions. These datasets are particularly valuable for uncovering E3 ligase–substrate relationships and prioritizing candidate regulators for functional validation. Second, single-cell approaches have enabled the resolution of cell-to-cell heterogeneity, allowing researchers to determine how molecular programs differ across cell populations, developmental states, or disease-associated microenvironments. Such analyses are especially important when bulk measurements mask rare but biologically significant subpopulations. Third, CRISPR-based loss-of-function or gain-of-function screens provide an unbiased framework to identify genes that regulate key phenotypes, including survival, proliferation, differentiation, and therapeutic response. When combined with multi-omics profiling, these screening strategies can help establish causal links between molecular alterations and functional outcomes. Although a detailed methodological review is beyond the scope of the present article, incorporating these emerging approaches is likely to deepen mechanistic insight and accelerate translational progress in the field.

## 5 Conclusion and Prospect

The novelty of the present review does not primarily lie in proposing entirely new molecular mechanisms, but rather in reorganizing existing ubiquitination-associated evidence within a temporally and biologically contextualized TNBC progression framework. Compared with conventional pathway-centered reviews, this stage-oriented perspective attempts to integrate tumor initiation, progression, treatment resistance, and immune adaptation into a unified interpretive model. This organization may help explain why certain ubiquitination regulators exhibit context-dependent or even divergent effects across different TNBC settings and may provide a conceptual basis for more temporally stratified therapeutic strategies. Ubiquitination should be viewed as a context-dependent regulatory system, rather than a static signaling entity. The same ubiquitination regulator may have divergent, or even opposing, effects depending on tumor stage, cellular context, and therapeutic pressure. This context dependency may explain why certain ubiquitination regulators exhibit divergent biological effects across different TNBC settings. Accumulating evidence suggests that while a limited number of recurrent regulatory hubs exert broader influence across TNBC progression, many additional pathways likely function in more subtype-specific or treatment-dependent contexts. Therefore, a stage-specific perspective offers a more comprehensive framework to reconcile seemingly conflicting findings in the literature and may help explain the limited efficacy of broadly applied targeting strategies. From a translational perspective, this model further suggests that therapeutic interventions targeting ubiquitination pathways should be tailored to tumor stage and molecular context, providing a rationale for the development of temporally stratified and precision-based treatment strategies in TNBC. This article systematically reviews the stage-specific regulatory roles of ubiquitination and UBL modifications in critical biological processes, including tumor initiation, progression, treatment resistance, and immune evasion in TNBC. Evidence indicates that the ubiquitination system, centered on E3 ligases and deubiquitinases, profoundly influences stemness maintenance, EMT, DNA damage repair, ferroptosis sensitivity, and immune checkpoint regulation by dynamically modulating the stability and activity of key proteins such as TLR4/NF-κB, TWIST1, PARP1, GPX4, and PD-L1. UBL modifications—including SUMOylation, NEDD8ylation, and ISGylation—synergistically contribute to genome stability, transcriptional control, and immune regulation, collectively forming the molecular framework underpinning TNBC malignancy.

Despite significant advancements in understanding the networks of ubiquitination and ubiquitin-like modifications in TNBC, several challenges persist that hinder their clinical translation. A major obstacle is the considerable heterogeneity among TNBC subtypes, which are associated with distinct molecular dependencies and highly variable therapeutic responses, thereby limiting the widespread applicability of ubiquitination-targeted strategies. This challenge is further exacerbated by the limited selectivity of currently available inhibitors targeting E3 ligases and deubiquitinating enzymes, which increases the risk of off-target effects and unintended systemic toxicity.

Simultaneously, the landscape of UBL modifications is inherently complex and dynamic, marked by extensive crosstalk with other post-translational modifications and signaling pathways, which poses substantial challenges for precise therapeutic intervention. In this context, the absence of robust, clinically validated predictive biomarkers constitutes a critical barrier, hindering effective patient stratification and limiting the accurate evaluation of treatment responses. Overcoming these interconnected challenges will be crucial for advancing ubiquitination-based therapeutic strategies toward clinically viable precision medicine in TNBC.

Current treatment strategies for TNBC exhibit considerable variation in the extent of clinical validation. Immune checkpoint inhibitors, such as anti-PD-1/PD-L1 antibodies, and PARP inhibitors have demonstrated robust clinical efficacy in selected patient populations and have either received regulatory approval or advanced to late-stage clinical trials. In contrast, most approaches targeting ubiquitination regulatory factors—including inhibitors of specific E3 ligases or deubiquitinating enzymes, as well as strategies modulating ferroptosis or PD-L1 ubiquitination dynamics—remain largely in preclinical or early translational stages. Although these emerging interventions have shown promising antitumor activity in experimental models, their safety, specificity, and clinical efficacy require further investigation. Consequently, before these therapies can be routinely applied in clinical practice, they must undergo rigorous, systematic, and standardized clinical evaluation. Looking forward, intervention strategies targeting the ubiquitination and UBL regulatory network hold substantial potential for clinical translation. Efforts should focus on constructing a TNBC-specific ubiquitinome and UBL modification map, systematically characterizing their dynamic changes across molecular subtypes and treatment pressures to provide a foundation for precise therapeutic targeting. Concurrently, there is an urgent need to develop highly selective small-molecule inhibitors of E3 ligases and DUBs and to explore their combination with immunotherapy, PARP inhibitors, antibody-drug conjugates (ADCs), and other therapeutic modalities to overcome existing treatment resistance. Furthermore, integrating multi-omics data with artificial intelligence is expected to enable dynamic assessment of protein homeostasis and prediction of therapeutic responses, shifting TNBC management from a “group-based” to an “individualized” approach. Multi-dimensional analysis and targeted intervention within the ubiquitination regulatory network are poised to open new avenues for overcoming treatment bottlenecks and achieving precise protein homeostasis modulation.

A major limitation of the proposed stage-specific framework is that it remains largely interpretive and synthesis-based. While it is grounded in available experimental and clinical literature, direct validation of stage boundaries, biomarker thresholds, and stage-tailored clinical utility is still insufficient. Future studies should establish reproducible biological definitions, test candidate biomarkers longitudinally, and evaluate whether stage-informed stratification improves prediction or treatment response. Although further prospective validation will be required to refine stage-associated biomarkers and therapeutic stratification strategies, the present framework provides an evidence-informed biological structure for integrating ubiquitination-associated mechanisms across different phases of TNBC progression.

## Data Availability

Not applicable.

## Abbreviations

TNBC	Triple-Negative Breast Cancer
ER	Estrogen Receptor
PR	Progesterone Receptor
HER2	Human Epidermal Growth Factor Receptor 2
EMT	Epithelial-Mesenchymal Transition
PTM	Post-Translational Modification
DUB	Deubiquitinating Enzyme
BCSC	Breast Cancer Stem Cell
PARP	Poly (ADP-Ribose) Polymerase
PARPi	PARP Inhibitor
PD-L1	Programmed Death-Ligand 1
PD-1	Programmed Cell Death Protein 1
ICI	Immune Checkpoint Inhibitor
UBL	Ubiquitin-Like
TME	Tumor Microenvironment
ADC	Antibody-Drug Conjugate
HRD	Homologous Recombination Deficiency
OS	Overall Survival
PFS	Progression-Free Survival
CPS	Combined Positive Score
EGF	Epidermal Growth Factor
EGFR	Epidermal Growth Factor Receptor
mTOR	Mechanistic Target of Rapamycin
NF-κB	Nuclear Factor Kappa-Light-Chain-Enhancer of Activated B Cells
HIF-1α	Hypoxia-Inducible Factor 1-Alpha
STAT3	Signal Transducer and Activator of Transcription 3
TGF-β	Transforming Growth Factor Beta
CRL	Cullin-RING Ligase
